# Rhodomyrtone Modulates Innate Immune Responses of THP-1 Monocytes to Assist in Clearing Methicillin-Resistant *Staphylococcus aureus*

**DOI:** 10.1371/journal.pone.0110321

**Published:** 2014-10-16

**Authors:** Sutthirat Srisuwan, Pongsri Tongtawe, Potjanee Srimanote, Supayang Piyawan Voravuthikunchai

**Affiliations:** 1 Department of Microbiology and Natural Products Research Center of Excellence, Faculty of Science, Prince of Songkla University, Songkla, Thailand; 2 Graduate Program in Biomedical Sciences, Faculty of Allied Health Sciences, Thammasat University, Pathumtanee, Thailand; Columbia University, United States of America

## Abstract

**Background:**

The increasing resistance of *Staphylococcus aureus* to conventional antibiotics poses a major health problem. Moreover, *S. aureus* can survive within phagocytes, thus evading some antibiotics and the innate immune response. Rhodomyrtone, a bioactive compound from the leaves of *Rhodomyrtus tomentosa*, possesses potent antibacterial activity against methicillin-resistant *S. aureus* (MRSA). This study was to investigate the immunomodulatory effects of rhodomyrtone on THP-1 monocytes in response to MRSA.

**Methods:**

THP-1 monocytes were stimulated with heat-killed MRSA, followed by treatment with rhodomyrtone. The cell pellets were prepared to detect pro-inflammatory molecules using real-time PCR. The supernatants were collected to assess nitric oxide production using Griess assay. Assays for phagocytosis and bacterial killing by THP-1 monocytes were performed to determine if they were affected by rhodomyrtone.

**Results:**

Expression of pro-inflammatory molecules including IL-1β, TNF-α, IL-6, and iNOS was enhanced in THP-1 monocytes stimulated with high doses of heat-killed MRSA (10^8^ to 10^9 ^cfu/ml). In contrast, monocytes stimulated with MRSA at lower doses (10^6^ to 10^7 ^cfu/ml) did not induce the expression of these cytokines. However, rhodomyrtone significantly increased the expression of pro-inflammatory mediators, IL-6 and iNOS in monocytes stimulated with heat-killed MRSA at low doses, and displayed some anti-inflammatory activity by reducing TNF-α expression in monocytes stimulated with heat-killed MRSA at high doses. Treatment with rhodomyrtone also significantly up-regulated the expression of the key pattern recognition receptors, TLR2 and CD14, in THP-1 monocytes stimulated with heat-killed MRSA at 10^6^ to 10^9^ cfu/ml, while heat-killed MRSA alone did not induce the expression of these molecules. The ability of rhodomyrtone to eliminate MRSA from the monocytes was observed within 24 h after treatment.

**Conclusion:**

Rhodomyrtone enhanced the expression of pattern recognition receptors by monocytes in response to MRSA. Increased expression of these receptors might improve MRSA clearance by modulating pro- and anti-inflammatory cytokine responses.

## Introduction

Methicillin-resistant strains of *Staphylococcus aureus* (MRSA) can express several potential virulence factors such as surface protein adhesins and toxins to invade and destroy host tissues. MRSA is a major cause of hospital-acquired infections and a frequent cause of infections associated with foreign bodies, including catheters and medical implants [1]. MRSA can overcome the host immune response by several mechanisms including interference with the production of pro-inflammatory cytokines, and the inhibition of phagocytosis and intracellular killing [2], [3].

The innate immune system plays an important role in host defense against pathogens, which it detects through specific pattern recognition receptors including toll-like receptors (TLR) and CD14 [4]. During infection, monocytes produce pro-inflammatory molecules, such as tumor necrosis factor-alpha (TNF-α), interleukin-1 beta (IL-1β), IL-6, and nitric oxide (NO) to destroy pathogens [5]. These molecules play crucial roles in innate immune responses such as phagocytosis and pathogen clearance. Enhancement of innate immunity by immunomodulatory agents could assist the host to recover from infection.

Various compounds from medicinal plants can be used to treat and prevent infections, and enhance immune function [6], [7]. However, only a few medicinal plant extracts have been shown to assist the host immune system to overcome infection with antibiotic-resistant strains of *S. aureus* [8], [9]. Rhodomyrtone, a bioactive compound from the leaves of *Rhodomyrtus tomentosa*, has pronounced antibacterial activity against several Gram-positive bacteria, including *S. aureus* and MRSA [10], [11]. The plant is also used in traditional medicine to treat urinary tract infection [12] and as antiseptic wash to clean wounds [13]. A recent study has shown that a methanolic leaf extract from *R. tomentosa* displays anti-inflammatory activity *in vitro* and *in vivo* [14]. However, no data have been reported on the effects of rhodomyrtone on innate immunity. The current study was undertaken to investigate the effects of rhodomyrtone on the production of pro-inflammatory cytokines, and the antibacterial activity of THP-1 monocytes in the presence or absence of MRSA.

## Materials and Methods

### Chemicals

RPMI 1640 medium, L-glutamine, penicillin/streptomycin, and TRizol were purchased from InvitroGen (California, USA). Foetal bovine serum (FBS) was obtained from HyClone (Cramlington, UK). Dimethyl sulphoxide (DMSO), phorbol 12-myristate 13-acetate (PMA), trypan blue solution, and carboxyfluorescein diacetate succinimidyl ester (CFSE) were purchased from Sigma-Aldrich (St. Louis, USA). Resazurin was obtained from Molecular Probes (Eugene, USA). All reagents were of purified grade.

### Purified rhodomyrtone

Rhodomyrtone was extracted from crude extract of *R. tomemtosa* leaf using column chromatography by our research group [15]. The purified rhodomyrtone was confirmed by nuclear magnetic resonance and mass spectrometry according to published method [16]. Rhodomyrtone was dissolved in 100% DMSO and stored at −20°C until further use.

### Preparation of MRSA suspension and heat-killed bacteria

A clinical isolate of MRSA (NPRC 001R) was kindly obtained from the Natural Product Research Center of Excellence (NPRCoE), Faculty of Science, Prince of Songkla University, Thailand. The bacterium was pre-cultured at 37°C for 24 h on blood agar. The single colony was transferred into 10 ml Trypticase soy broth and grown at 37°C with shaking to mid-exponential phase. The culture was centrifuged at 10,000×*g* at 4°C for 10 min. The cell pellet was collected, washed twice with phosphate buffered saline (PBS), pH 7.4 and resuspended in RPMI 1640 medium containing 10% FBS. The bacterial suspension was adjusted to approximately 1×10^9^ cfu/ml (OD_600_ = 1) and confirmed by counting colonies on blood agar. For some studies, bacteria were heat-killed at 90°C for 30 min in a water bath.

### Cell culture

THP-1 human monocyte cell line (ATCC TIB-202) was maintained in RPMI 1640 medium supplemented with 10% FBS, 2 mM L-glutamine, 100 units/ml of penicillin and 100 µg/ml of streptomycin at 37°C in humidified incubator with 5% CO_2_. The medium was changed every two days. For macrophage differentiation, 10^6^ cells/ml were plated in fresh RPMI 1640 medium in a 24-well plate and activated with PMA at final concentration of 10 ng/ml for 24 h.

### Cell viability assay

THP-1 cells were adjusted to 1×10^5^ cells/ml and 100 µl aliquots were seeded into each well of a 96-well plate and incubated at 37°C for 3 h in a CO_2_ incubator. Rhodomyrtone at concentrations ranging from 0.195 to 50 µg/ml was added; DMSO at final concentration of 0.625% was used as a negative control. After 20 h incubation, 10 µl resazurin (final concentration 50 µg/ml) was added to the THP-1 cells and incubated for 4 h. The fluorescence intensity of viable cells at 590 nm emission (FI_590_) was measured using a microplate reader (Varioskan Flash). Rhodomyrtone at concentration up to 1.56 µg/ml was not toxic to THP-1 cells (data not shown).

### Rhodomyrtone treatment

THP-1 cells (1×10^6^ cells/ml) were cultured in a 24-well plate for 4 h and stimulated with heat-killed MRSA at cell concentrations ranging from 1×10^6^ to 1×10^9^ cfu/ml. After 4 h incubation, the cell culture was treated with rhodomyrtone at 0.39 to 1.56 µg/ml. Lipopolysaccharide (LPS) from *Salmonella* Typhi was prepared by the hot phenol-water extraction and was used as positive control. The treated monocytes were incubated for 6 h before measuring the pro-inflammatory cytokines, TLR2, and CD14 by quantitative real-time polymerase chain reaction (qRT-PCR). Levels of nitric oxide were measured at 24 h as described below.

### Quantitative real-time polymerase chain reaction (qRT-PCR)

Total RNA was extracted from THP-1 cells using TRizol reagent according to the manufacturer’s instructions (Life Technologies). Total RNA was used for one-step real-time PCR amplification using Brilliant II SYBR Green qRT-PCR one-step Master Mix Kit (Stratagene, USA) and a Rotor gene 3000 (Corbett Research) machine. The PCR primers used are listed in Table 1. The reaction mixture was incubated at 42°C for 60 min and then denatured at 95°C for 10 min. The PCR samples were amplified 45–55 cycles under the following conditions: for the Bax, caspase-3, TNF-α, and nitric oxide synthase (iNOS) genes, denaturation at 95°C for 15 s, annealing at 62°C for 60 s, and extension at 72°C for 60 s; for the IL-1β gene, denaturation at 95°C for 30 s, annealing at 56°C for 60 s, and extension at 72°C for 30 s; for the IL-6, TLR2, and CD14 genes, denaturation at 95°C for 15 s, annealing at 60°C for 60 s, and extension at 72°C for 60 s. The PCR conditions for the β-actin gene (control) varied in accordance with the gene being assayed. β-actin was used as housekeeping gene and to confirm quality of reverse-transcripted cDNA.

**Table 1:** Primers for real-time PCR.

mRNA	Forward primer (5′ to 3′)	Reverse primer (5′ to 3′)
Bax	TGGAGCTGCAGAGGATGATTG	GAAGTTGCCGTCAGAAAACATG
Capase-3	CAGTGGAGGCCGACTTCTTG	TGGCACAAAGCGACTGGAT
IL-1β	GACACATGGGATAACGAGGC	ACGCAGGACAGGTACAGATT
TNF-α	GCCCAGGCAGTCAGATCATC	CGGTTCAGCCACTGGAGCT
IL-6	TGACAAACAAATTCGGTACATCCT	TCTGCCAGTGCCTCTTTGCT
iNOS	GCTGTATTTCCTTACGAGGCGAAGAA	CTTGTTAGGAGGTCAAGTAAAGGGC
TLR2	GGCCAGCAAATTACCTGTGTG	AGGCGGACATCCTGAACCT
CD14	CGCTCCGAGATGCATGTG	TTGGCTGGCAGTCCTTTAGG
β-actin	GAGCGGGAAATCGTGCGTGACATT	GAAGGTAGTTTCGTGGATGCC

The expression levels of genes were quantified using the ΔΔC_T_ method to determine fold change in gene expression (2^−ΔΔCT^). The results of each sample were expressed as log_2_ fold-change in gene expression, and were accepted if the expression of inclusion criteria (Bax and casp3 apoptosis genes) exhibited a log_2_ fold change of ±1. For the test samples, a statistically significant change in expression of ≥1−log_2_ fold was taken as up-expression, and a ≤1−log_2_ fold change was down-expression.

### Nitric oxide analysis

The production of nitric oxide (NO) was investigated by measuring nitrite in the culture supernatants using the Griess reagent according to the manufacturer’s recommendations (Promega). THP-1 cells were stimulated with heat-killed MRSA, followed by rhodomyrtone for 24 h. After incubation, the cells were centrifuged and the supernatant was collected. Fifty-microliter aliquots were incubated with Griess reagent (sulfanilamide solution and NED solution, respectively) in a 96-well ELISA plate. The samples were incubated at room temperature for 10 min in the dark, and absorbance was measured at 540 nm within 30 min using a microplate reader (Varioskan Flash). Nitrite concentrations were determined from a standard curve prepared by assaying serially-diluted sodium nitrite.

### Assay for CD14 surface marker

THP-1 cells (1×10^5^ cells/ml) were treated with rhodomyrtone at 0.39 and 1.56 µg/ml for 6 h. The treated cells were harvested, washed, and resuspended in RPMI 1640 medium. The density of the treated cells, non-stimulated THP-1 cells, and PMA-stimulated THP-1 cells was adjusted to 1×10^5^ cells/ml. Primary human monocytes were used as positive control. For detection of CD14 cell surface marker, monoclonal mouse anti-human antibodies IgG2κ CD14-PE (M5E2, BioLegend) at a final concentration of 0.05 µg/ml were incubated with the samples at 4°C for 30 min in the dark. The samples were then washed with ice cold PBS, harvested, resuspended in PBS, and fixed with 1% paraformadehyde. Cells were then enumerated by using flow cytometry (Beckman Coulter Quanta SC). All data were analyzed with InCyte Version 2.6 program.

### Intracellular survival assay

Intracellular survival assays were adapted from a published procedure [17]. The killing of MRSA by THP-1 monocytes was measured by determining the reduction in the number of viable bacteria in the monocytes. THP-1 monocytes and THP-1-derived macrophages (1×10^6^ cells/ml) were mixed with MRSA (1×10^6^ cfu/ml) at multiplicity of infection (MOI) of 1 in a 24-well plate and incubated for 30 min in 5% CO_2_. The wells were washed three times with PBS by centrifugation at 400×*g* for 4 min. Gentamicin at a final concentration of 300 µg/ml was then added to kill any remaining extracellular bacteria for 1 h. Infected cells were incubated in the presence of rhodomyrtone at 0.0975 to 1.56µg/ml. At specific time points, these cells were washed three times in PBS and resuspended in 0.02% Triton X-100 for 30 min to lyse the THP-1 cells. Viable bacteria in cell lysates were plated on Luria agar and incubated overnight. The intracellular survival index (SI_t_) was calculated according to the formula: SI*_t_* = [(cfu/mL)_t_/(cfu/mL)_0_]×100, where SI_t_ indicates the percentage of viable intracellular bacteria (cfu) at a specific time compared with at time 0.

### Phagocytosis assay

For the phagocytosis assay, MRSA were labeled with CFSE as follows: cells (1×10^9^ cfu/ml) were cultured in Luria broth and centrifuged. The pellet was washed with PBS, centrifuged, and resuspended in PBS containing 5% FBS and CFSE at a final concentration of 10 µM. Samples were then incubated for 10 min at room temperature in the dark. The bacterial suspension was washed twice with a 10X volume of PBS supplemented with 5% FBS. The labeled bacteria were then fixed with 1% paraformaldehyde in PBS, washed with PBS supplemented with 5% FBS. The washed bacterial cells were resuspended in medium containing 10% FBS and fluorescence was determined by flow cytometry. More than 90% of bacteria were labeled with CFSE by this method (data not shown).

THP-1 monocytes (1×10^6^ cells/ml) were treated with rhodomyrtone at 1.56 µg/ml. The treated monocytes and labeled bacteria were co-incubated at ratios of 1∶100 and 1∶1,000 (monocytes:bacteria) in media containing 10% FBS. After 30 and 60 min the assay was stopped by washing with 1 ml ice-cold PBS. The cells were fixed by adding PBS containing 1% paraformaldehyde, and analysed by using flow cytometry (Beckman Coulter FC500). Phagocytosis of labeled bacteria was analyzed with DaCS Version 1.2.03 from the fluorescence intensity per cell.

### Statistical analysis

Unless otherwise stated, experimental results are presented as the mean ± SEM of the results at least three independent experiments in performed in triplicate samples. Paired two-tailed Student’s t-test was used to compare the test and control groups. P values of <0.05 were taken to indicate statistical significance.

## Results and Discussion

### Rhodomyrtone enhances IL-6 and decreases TNF-α expression in THP-1 monocytes stimulated with MRSA

Upon infection with *S. aureus*, a pro-inflammatory cascade is activated leading to the production of inflammatory molecules. However, the bacteria can protect themselves against innate immune responses by altering signal transduction and reducing the expression of pro-inflammatory molecules. In addition, biofilm-forming staphylococci and intracellular bacteria can evade host immune cells resulting in weak production of pro-inflammatory cytokines and chemokines [18], [19], [20].

**Figure 1:**
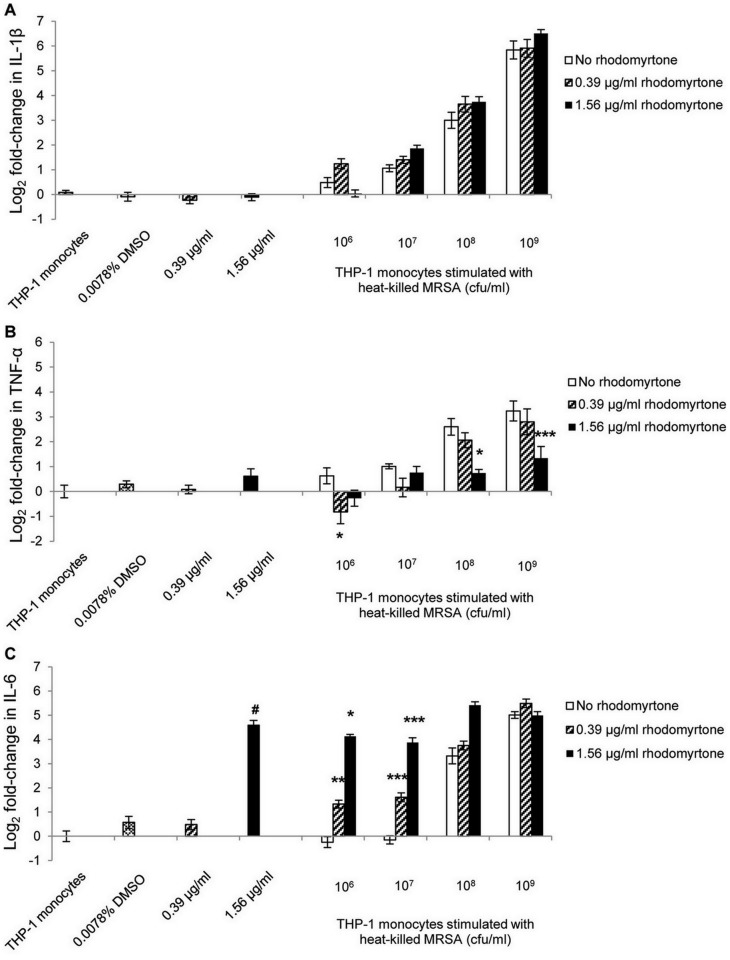
Pro-inflammatory cytokine expression by THP-1 monocytes in response to MRSA stimulation, followed by rhodomyrtone treatment. The monocytes were incubated with heat-killed MRSA at 10^6^, 10^7^, 10^8^, and 10^9^ cfu/ml, followed by rhodomyrtone treatment (0.39 and 1.56 µg/ml) for 6 h. Monocytes were collected and RNA was isolated for the measurement of mRNA levels of IL-1β (A), TNF-α (B), and IL-6 (C) by real-time PCR. Gene expression was calculated relative to β-actin gene and expressed as log_2_ fold change. The data are the mean ± SEM from three independent experiments and two technical replicates. p values were calculated with reference to no rhodomyrtone values at corresponding doses. * p<0.05, ** p<0.01, *** p<0.001. # p<0.001, compared with the unstimulated monocytes.

In this study, we stimulated THP-1 monocytes with heat-killed MRSA and found that monocytes stimulated with heat-killed MRSA at 10^8^ to 10^9^ cfu/ml increased their expression of IL-1β, TNF-α and IL-6 mRNA in a dose dependent manner (Figure 1). The data suggested that pro-inflammatory cytokines were induced by heat-inactivated bacteria at high concentrations (10^8^ to 10^9^ cfu/ml). The bacterial cells activate downstream cytokine cascade leading to induction of a broad spectrum of cytokines. A complete cell wall is necessary to be an inducer in pro-inflammatory cytokine expression [17], [21]. Immune response to *S. aureus* requires a large quantity of peptidoglycan at concentrations ranged from 10 to 100 µg/ml which are equivalent to 6×10^7^ and 6×10^8^ cfu/ml of the bacterial cells, respectively [21]. Cytokines, such as IL-1β, TNF-α, and IL-6 are important effectors in host defense against *S. aureus* [22]. Many medicinal compounds are said to act as immunostimulators that enhance immune responses, partly by stimulating cytokine production [23], [24], [25]. To determine if rhodomyrtone can act as an immunomodulator, THP-1 monocytes were treated with rhodomyrtone at 0.195 to 1.56 µg/ml for 6 h. After treatment, expression of IL-1β, TNF-α, and IL-6 mRNA was determined by real-time PCR. The results demonstrated that the monocytes treated with rhodomyrtone exhibited no change in IL-1β or TNF-α mRNA expression. In contrast, rhodomyrtone induced IL-6 expression in a dose dependent manner. Examination of apoptosis gene expression (Bax and casp3) showed that the induction of IL-6 was not related to cellular toxicity (data not shown).

To determine if rhodomyrtone can act as an immunomodulator on MRSA-activated THP-1 monocytes, the monocytes were activated by heat-killed MRSA, followed by treatment with rhodomyrtone. The results showed that rhodomyrtone had no effect on IL-1β expression in MRSA-stimulated monocytes (Figure 1A). In contrast, treatment with rhodomyrtone at 1.56 µg/ml significantly reduced TNF-α expression induced by MRSA at 10^8^ and 10^9^ cfu/ml (p<0.05, p<0.001, respectively; Figure 1B). Monocytes stimulated with heat-killed MRSA at low doses (10^6^ and 10^7^ cfu/ml), followed by treatment with rhodomyrtone (1.56 µg/ml) showed a significant increase in IL-6 mRNA (p<0.05, p<0.001, respectively; Figure 1C). In addition, rhodomyrtone at a low concentration (0.39 µg/ml) significantly enhanced the immunomodulatory activity of monocytes stimulated with low doses of MRSA (p<0.01, p<0.001, Figure 1C). Interestingly, however, the expression levels of IL-6 were not higher than controls when THP-1 monocytes were stimulated with MRSA at high doses (10^8^ and 10^9^ cfu/ml; Figure 1C). These findings suggest that rhodomyrtone at 0.39 µg/ml could act synergistically with host defenses to induce IL-6 expression in monocytes stimulated with MRSA in low numbers, similar to those encountered during infection. By contrast, the increased expression of IL-6 by rhodomyrtone at 1.56 µg/ml in THP-1 cells treated with MRSA at low doses may be due to the effect of rhodomyrtone alone.

IL-6 plays a role in both the activation and suppression of inflammatory responses. IL-6 also contributes to antimicrobial activity in mice infected with Gram-positive bacteria by suppressing the production of TNF-α [26]. Furthermore, IL-6 can induce monocytes to produce low levels of antimicrobial peptides, such as hepcidin [27]. Medicinal agents may enhance immune responses by affecting IL-6 production [28], [29], [30], [31]. For example, β-glucan from *Aureobasidium pullulans* [31] and a crude extract from *Aloe secundiflora* [32] can induce IL-6 production and increase antibody levels.

TNF-α makes an important contribution to the development of chronic inflammation. Anti-TNF-α drugs and antibodies are increasingly being used therapeutically to reduce excessive inflammation in autoimmune diseases [33]. Reducing TNF-α production with drugs can protect from *S. aureus*-induced sepsis [34].

Ideally, immunomodulators should enhance local host defenses against bacterial infections without increasing inflammation. PGG-Glucan, a soluble beta-(1,6)-branched beta-(1,3)-linked glucose homopolymer, demonstrates good activities against multidrug-resistant *S. aureus*, which correlated with an enhanced oxidative burst activity in infected neutrophils [35]. Interestingly, our results suggested that rhodomyrtone at anti-MRSA concentration (1.56 µg/ml, which is equivalent to 2MIC)) enhances IL-6 and suppresses TNF-α expression in monocytes stimulated with MRSA. Possibly, rhodomyrtone plays a protective role in MRSA-stimulated monocytes by stimulating the production of IL-6 and suppressing of TNF-α expression. IL-6 could inhibit excessive inflammation by reduction of IL-1 and TNF production [36]. Our data indicate that rhodomyrtone at anti-staphylococcal concentrations could be used as an immunomodulator for the treatment of MRSA infections.

### Rhodomyrtone induces iNOS mRNA and nitric oxide production in THP-1 monocyte cells

**Figure 2:**
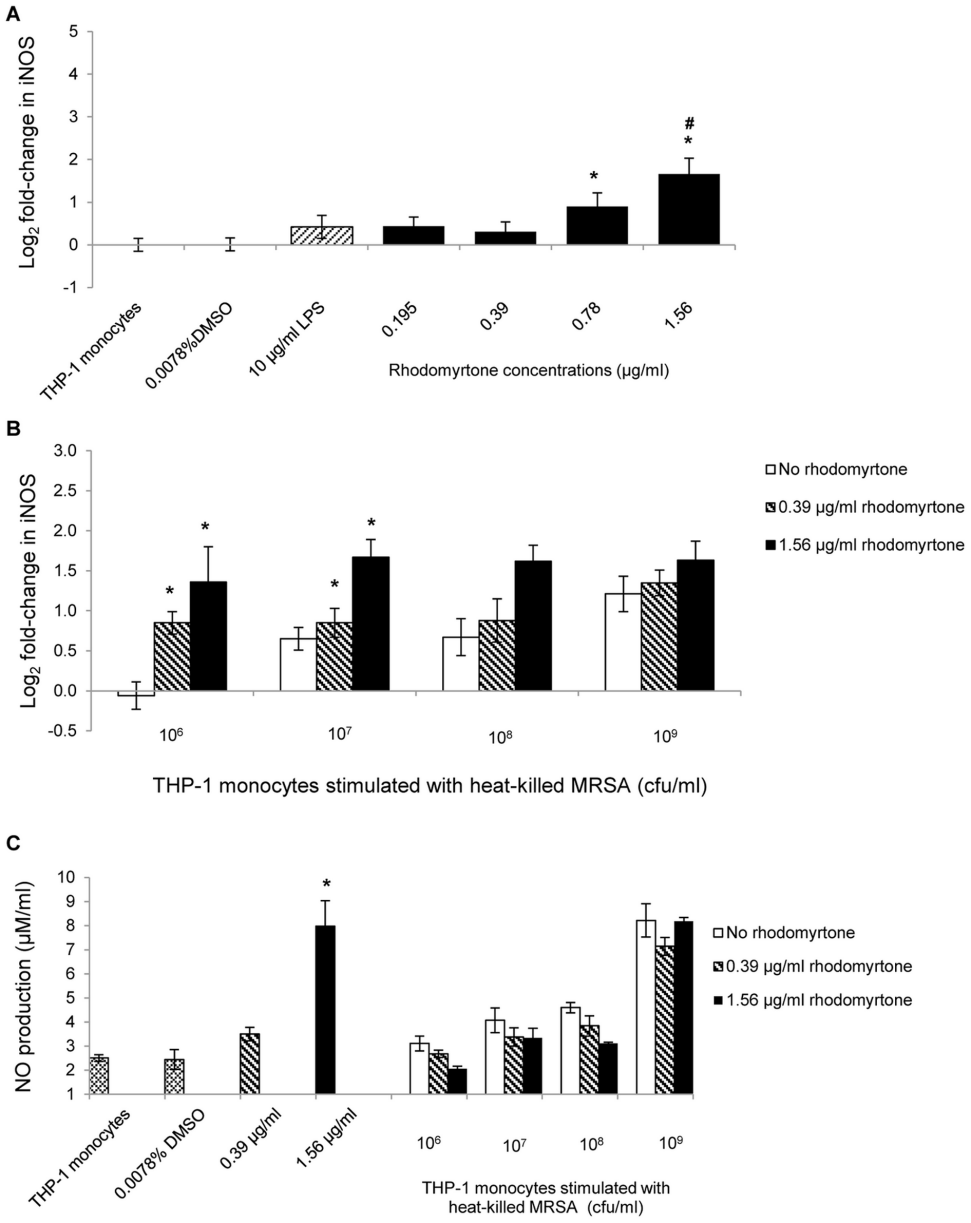
Effect of rhodomyrtone on iNOS expression in THP-1 monocytes. THP-1 cells were treated with rhodomyrtone at concentrations from 0.195 to 1.56 µg/ml for 24 h. Cells were collected and RNA was extracted for the determination of iNOS mRNA levels using real-time PCR (A). Gene expression was calculated relative to β-actin mRNA and the untreated monocytes and expressed as log_2_ fold change (ΔΔCt). Data are the mean ± SEM from three independent experiments and two technical replications. * p<0.05, compared with the untreated controls. # p<0.05, compared with the monocytes treated with LPS. THP-1 monocytes were stimulated with heat-killed MRSA at 10^6^, 10^7^, 10^8^, and 10^9^ cfu/ml, followed by rhodomyrtone (0.39 or 1.56 µg/ml) for 24 h. Cells were collected and RNA was extracted for the determination of iNOS mRNA levels by real-time PCR (B). * p<0.05, compared to no rhodomyrtone controls. The supernatant was collected and used to measure NO production using the Griess assay (C). Data are present the mean ± SEM from three independent experiments and two technical replicates. * p<0.05, compared with untreated monocytes.

The studies described above showed that rhodomyrtone affected selective early pro-inflammatory molecules in THP-1 monocytes. We also wanted to see if rhodomyrtone had an effect on the production of late inflammatory molecules. To this end we determined levels of iNOS expression in THP-1 monocytes treated with rhodomyrtone for 24 h. Rhodomyrtone at 0.78 and 1.56 µg/ml significantly increased iNOS mRNA expression by 0.90- and 1.66−log_2_ fold, respectively, compared with untreated monocytes (p<0.05, Figure 2A). In addition, rhodomyrtone at 1.56 µg/ml also led to a significant increase in NO production by 7.89 µM (p<0.05, Figure 2C).

iNOS expression results in the production of NO which reduces the viability of Gram-positive and Gram-negative bacteria, especially intracellular bacteria [37], [38]. The bactericidal action of NO against antibiotic-resistant pathogens, including MRSA, may be enhanced by its ability to interfere with biofilm formation [39].

Medicinal compounds that have been demonstrated to boost immune functions by inducing iNOS expression include tannins and related compounds which up-regulate iNOS expression in infected macrophages [40]. To determine if rhodomyrtone can enhance iNOS production by THP-1 monocytes stimulated with MRSA, we pre-incubated monocytes with heat-killed MRSA and then treated them with rhodomyrtone for 24 h. We found that rhodomyrtone at 1.56 µg/ml significantly enhanced iNOS mRNA expression, compared with control monocytes treated with low doses of heat-killed MRSA alone (p<0.05, Figure 2B). Rhodomyrtone at a lower concentration (0.39 µg/ml) also enhanced iNOS expression in monocytes stimulated with MRSA. Although our results suggest that rhodomyrtone may enhance iNOS expression in monocytes stimulated with MRSA, the treatment did not significantly iNOS activity at the protein level at 24 h (p>0.05, Figure 2C).

### Rhodomyrtone enhances the expression of TLR2 and CD14 receptors on THP-1 monocytes stimulated with MRSA

**Figure 3:**
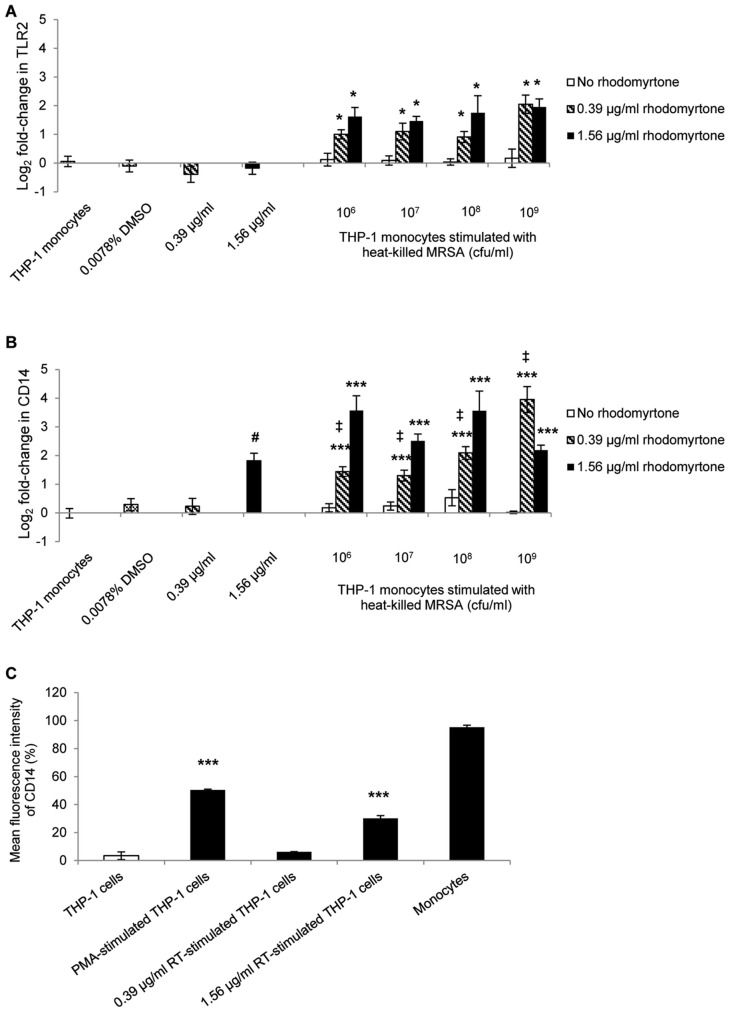
Effect of rhodomyrtone on the expression of TLR2 and CD14 by THP-1 monocytes stimulated with MRSA. THP-1 monocytes were stimulated with heat-killed MRSA at 10^6^, 10^7^, 10^8^ or 10^9^ cfu/ml, followed by treatment rhodomyrtone (0.39 or 1.56 µg/ml) for 6 h. Cells were collected and RNA was assayed for the amount of mRNA for TLR2 (A) and CD14 (B) using real-time PCR. Data are the mean ± SEM from three separate experiments and two technical replicates. * p<0.05, ** p<0.01, *** p<0.001, compared with no rhodomyrtone controls. ‡ p<0.01, compared with 0.39 µg/ml rhodomyrtone treatment alone. # p<0.001, compared with untreated monocytes. Median fluorescent intensity (MFI) of CD14 (C) expressed on the cell surface of THP-1 cells untreated or treated with PMA or rhodomyrtone (0.39 or 1.56 µg/ml) for 6 h. *** p<0.001, compared with the untreated monocytes.

Macrophages are an important first line of defense against Gram-positive bacteria due in part to the expression of innate immune receptors such as TLR2. Cell wall components of *S. aureus* then interact with TLR2 leading to the expression of pro-inflammatory molecules. Initiation of innate immunity requires cooperation of TLR2 with CD14 to enhance induction of inflammatory activity [41]. In the present study, we tested the effect of rhodomyrtone on TLR2 and CD14 mRNA expression in THP-1monocytes stimulated with heat-killed MRSA and treated with rhodomyrtone. The results demonstrated that heat-killed MRSA failed to stimulate TLR2 (Figure 3A) or CD14 expression in monocytes (Figure 3B). The nature of pathogen-associated molecular pattern molecules in *S. aureus* is not entirely clear, although there is evidence that the induction of expression of TLR2 and CD14 by *S. aureus* is dependent on their bacterial cell walls and toxins [2], [42]. On the other hand, many different bacteria can modify their cell wall as a way of evading the immune response [43].

Several plant-derived compounds can act as TLR ligands and modulate immune responses. Melanin from *Nigella sativa* binds to TLR4 leading to the production of IL-8 and IL-6 [23], [24]. In future, immunostimulatory compounds of this type could be used as vaccine adjuvants to enhance immune responses.

The expression of TLR2 and CD14 is an indicator of monocyte activation, although CD14 expression is very low in THP-1 cells [44]. Our results, indicated that monocytes treated with rhodomyrtone at 1.56 µg/ml for 6 h significantly increase CD14 expression at the mRNA and protein levels, compared with untreated cells (p<0.05, p<0.001, respectively; Figures 3B and C). In contrast, rhodomyrtone did not stimulate TLR2 expression on the monocytes (Figure 3A). Nevertheless, monocytes stimulated with heat-killed MRSA, followed by rhodomyrtone at 0.39 and 1.56 µg/ml significantly enhanced transcriptional expression of both TLR2 and CD14 when compared with heat-killed MRSA alone (p<0.05, p<0.001, Figures 3A, B). The results indicate that rhodomyrtone can induce activation of promonocytic cells into activated monocytes through the induction of selected cytokines, iNOS, and CD14 expression.

### Rhodomyrtone enhances intracellular killing of MRSA by THP-1 cells

**Figure 4:**
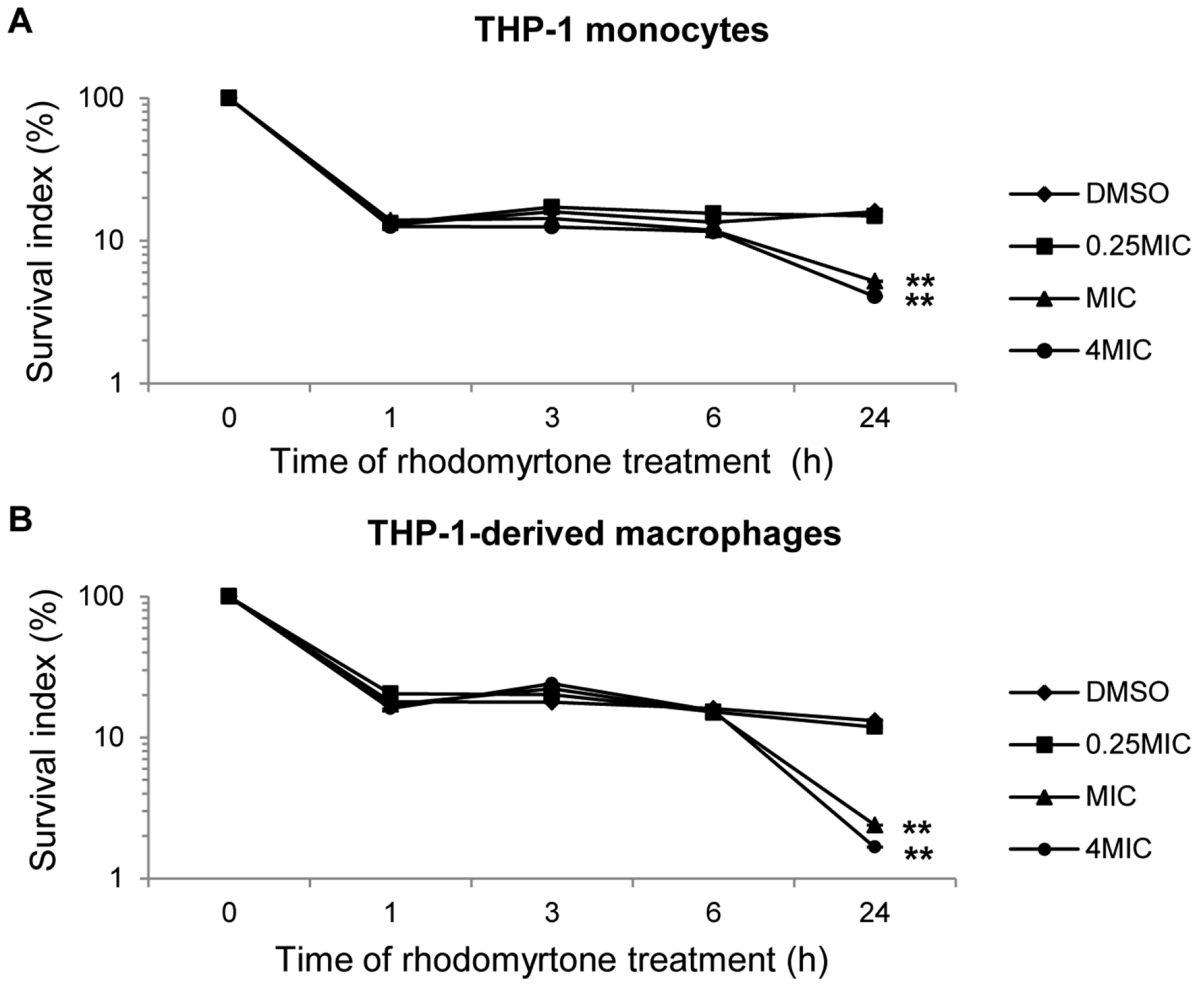
Effect of rhodomyrtone on the bactericidal activity of phagocytes. THP-1 monocytes (A) and PMA-activated THP-1 monocytes (B) were incubated with MRSA at the multiplicity of infection of 1 for 30 min. Cells were treated with rhodomyrtone at 0.0975 (0.25MIC), 0.39 (1MIC), or 1.56 (4MIC) µg/ml for the times indicated. Negative-control cells were treated with the vehicle (0.0078% DMSO) alone. Data are the mean ± SD of 3 independent experiments. ** p<0.01, compared with DMSO controls.

A recent study showed that *S. aureus* can survive within THP-1 macrophages for at least 24 h [45]. The intracellular bacteria may contribute to treatment failures with antibiotics that achieve low intracellular concentrations [46]. The aim of this part of our study was to investigate the effects of rhodomyrtone on the bactericidal capacity of human THP-1 monocytes and monocyte-derived macrophages. The monocytes and macrophages were incubated with MRSA at multiplicity of infection of 1, followed by treatment with rhodomyrtone at 0.0975, 0.39, and 1.56 µg/ml (0.25, 1, and 4MIC, respectively). After 24 h, rhodomyrtone at MIC and 4MIC had significantly reduced the percentages of viable MRSA in monocytes, compared with the control with no rhodomyrtone treatment (p<0.01, Figure 4A). Similarly, macrophages treated with rhodomyrtone were able to kill MRSA to a significantly greater extent than the controls (p<0.01, Figure 4B). Our data indicate that rhodomyrtone at MIC and 4MIC could enhance the bactericidal capacity of both monocytes and monocyte-derived macrophages. Together with our other findings, these data suggest that the mechanism of bacterial killing might involve direct activation of promonocyte THP-1 cells, leading to the enhancement of innate immune responses, such as increased production of IL-6, which in turn induces antimicrobial peptide production by monocytes [27], [47]. The antimicrobial peptide has been successful in antimicrobial activity and immunomodulatory effects in monocytes [27]. Futhermore, our results indicated that rhodomyrtone could directly modulate innate immune effectors in THP-1 monocytes leading to enhanced MRSA clearance.

### Rhodomyrtone does not enhance the phagocytic activity of THP-1 monocytes

**Figure 5:**
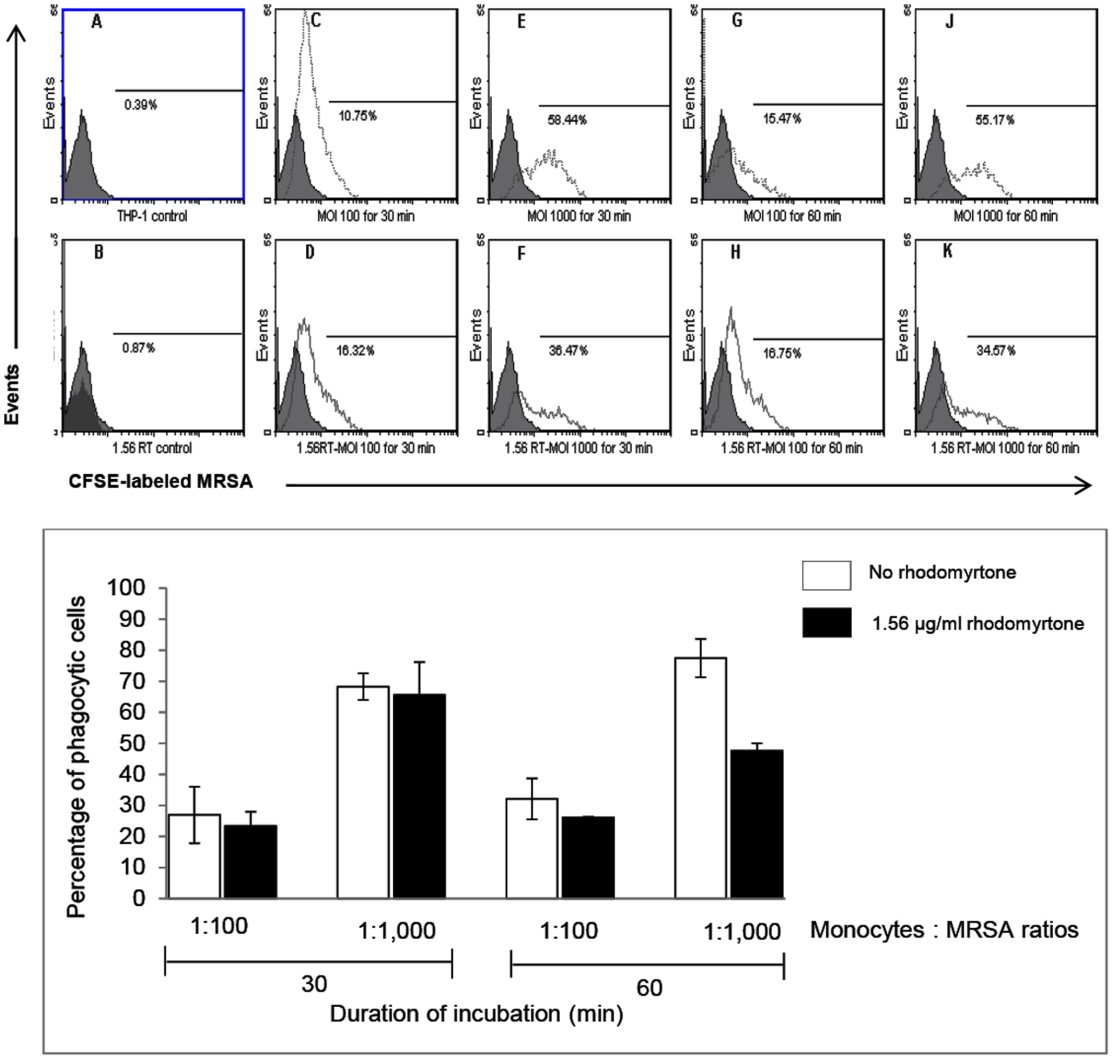
Phagocytic activity of THP-1 monocytes determined by flow cytometry. THP-1 monocytes were treated with or without 1.56 µg/ml rhodomyrtone, after which they were incubated with CFSE-labeled killed MRSA at ratios of 1∶100 and 1∶1,000 (monocytes:bacteria) for 30 or 60 min. Experimental data are shown in the top panel and summarized in the bottom panel, where data are the mean ± SD percent phagocytic activity of monocytes, determined from the mean fluorescence intensity compared to control monocytes.

During bacterial infection, phagocytic cells produce high levels of reactive oxygen species and nitrogen intermediates that contribute to bacterial killing. Bacterial cell wall components, such as peptidoglycan are keys inducers of the innate immune response and macrophage activation [17], [42], [43]. In this study, we observed that rhodomyrtone did not affect phagocytic activity of THP-1 monocytes exposed to killed MRSA at concentrations of 10^8^ to 10^9^ cfu/ml, which contain around 11 to 110 µg/ml of peptidoglycan, respectively (data not shown). Specifically, pre-treatment of monocytes with rhodomyrtone, followed by incubation with dead CFSE-labeled MRSA at 1∶100 and 1∶1000 ratios (monocytes: bacteria) demonstrated no difference of mean fluorescence intensity, compared with unstimulated monocytes at 30 and 60 min (p>0.05, Figure 5). The fact that the bacterial cells were not opsonized, however, may have affected our findings. Nevertheless, our data indicate that enhancement of intracellular killing by rhodomyrtone is independent of non-opsonic phagocytosis.

We also found that heat-killed MRSA increased pro-inflammatory cytokines and iNOS expression in THP-1 monocytes in a dose dependent manner, without changing the expression of TLR2 and CD14. Both TLR2 and CD14 are key receptors in innate immune response to Gram-positive bacterial infection. TLR2 requires CD14 to completely activate downstream cytokine cascade in response to the infection. Activation of these receptors can lead to induction of antimicrobial activity.

Rhodomyrtone alone also stimulated pro-inflammatory molecules such as IL-6 and iNOS, as well as CD14. Rhodomyrtone also enhanced the expression of pro-inflammatory cytokines, especially IL-6, and the pattern recognition receptors, TLR2 and CD14, in THP-1 monocytes stimulated with heat-killed MRSA. Treatment of bacteria-stimulated monocytes with rhodomyrtone also enhanced their ability to kill bacteria, while down-regulating expression of TNF-α. Together, our findings suggest that rhodomyrtone could be used as immunomodulatory agent to enhance innate immune responses against bacterial infections.
